# Branching out: mobilizing community assets to support the mental health and wellbeing of children in primary schools

**DOI:** 10.3389/fpubh.2024.1386181

**Published:** 2024-06-28

**Authors:** Anna Dadswell, Hilary Bungay, Faye Acton, Nicola Walshe

**Affiliations:** ^1^School of Allied Health and Social Care, Faculty of Health, Medicine and Social Care, Anglia Ruskin University, Chelmsford, United Kingdom; ^2^School of Allied Health and Social Care, Faculty of Health, Medicine and Social Care, Anglia Ruskin University, Cambridge, United Kingdom; ^3^Veterans and Families Institute, Faculty of Health, Medicine and Social Care, Anglia Ruskin University, Chelmsford, United Kingdom; ^4^Institute of Education, Faculty of Education and Society, University College London, London, United Kingdom

**Keywords:** arts-in-nature, children’s mental health, creative health, mobilizing community assets, primary schools, public health intervention, school staff, volunteers

## Abstract

**Introduction:**

Mobilizing existing creative, cultural and community assets is seen as a crucial pathway to improving public health. Schools have been identified as key institutional community assets and arts-in-nature practice has been shown to promote children’s mental health. The ‘Branching Out’ research investigated how an established arts-in-nature practice called ‘Artscaping’ could be scaled up through the mobilization of community assets including school staff and local volunteers to reach more children in primary schools.

**Methods:**

The Branching Out model was piloted in six primary schools across Cambridgeshire with ‘Community Artscapers’ delivering 1.5-h Artscaping sessions with children outdoors for 8 weeks. Interviews were conducted with 11 Community Artscapers (six school staff and five volunteers) and four school leaders reflecting on their experiences of the Branching Out model and the data was subject to a reflexive thematic analysis.

**Results:**

The findings presented here discuss themes relating to mobilizing community assets, including framing the opportunity, recruiting and sustaining volunteers, training and supporting Community Artscapers, and tensions in roles and responsibilities. They also cover impacts for the children, including mental health provision, freedom in creativity and being outside, personal development, emotional impacts, and social connection, as well as impacts for the Community Artscapers, including making a difference, emotional wellbeing, personal and professional development, and connection and community.

**Discussion:**

These findings are considered in terms of their alignment with public health policy drivers and the potential for the Branching Out model to become replicable and self-sustaining across schools to promote children’s mental health as a public health intervention.

## 1 Introduction

Children’s mental health is a pressing public health concern that has only been exacerbated in recent years by the COVID-19 pandemic, the rising cost of living, and the impact of global events. In England in 2023, 20.3% of children aged 8–16 years had a probable mental health disorder (1). Subjective wellbeing – particularly in relation to appearance, friends, school, and life as a whole – has also declined in the period from 2009–10 to 2019–20 (2). Furthermore, research has shown persistent inequalities in children’s mental health, and it has been suggested that the mental health gap between advantaged and disadvantaged children is growing (3, 4). For example, research has found that on starting primary school, children from the most deprived backgrounds had a higher prevalence of mental health difficulties compared with those from affluent backgrounds, and this disparity widened dramatically over the first three years of school (5). Poor mental health has long-term impacts on academic performance, social relationships, and overall quality of life, yet many children and young people do not receive adequate support (6, 7). Data suggests that 48.9% of parents of children with a probable mental health disorder sought help and advice from health services compared to 73.6% who sought help and advice from educational services (1), with increased pressure on the National Health Service (NHS) mental health services leading to long waiting lists and poor experiences for children and young people (8–10).

One approach to supporting children’s mental health and wellbeing is through arts-in-nature practice. A growing body of research demonstrates that participatory arts activities can improve both wellbeing and social inclusion [e.g., (11, 12)]. A review by Zarobe and Bungay (13) found that participating in arts activities had a positive impact on children and young people’s self-confidence, self-esteem and sense of belonging, factors which have been linked to resilience and mental wellbeing. A more recent review by Birell et al. (14) looked at the impact of arts-inclusive programs on young children’s mental health and wellbeing and identified emerging evidence of positive impacts for children aged 0–6 years. However, both these reviews highlighted the lack of high-quality research in this area and called for further research to address this. Meanwhile, contact with nature has considerable benefits for wellbeing (15, 16). This is particularly significant in a context within which 60% of children in England have spent less time outdoors since the COVID-19 pandemic (17), with a recent survey revealing that the rate of playing outdoors has been steadily falling for decades (18). Evidence suggests that combining the two and undertaking creative arts activities in nature has the potential to support children’s mental health and wellbeing in community and school settings. Moula et al. (19) undertook a systematic review in which they synthesized arts-based interventions delivered in nature to over 600 children and young people across five countries. They found evidence that arts-in-nature can: provide multi-sensory stimuli for children to connect with nature, understand environmental issues and explore ways to prevent environmental disasters, thus addressing eco-anxiety; engage children who might be disinterested about environmental issues, disengaged with educational programs, or feel excluded from existing programs; and enhance social connectedness, and structural and social capital, thereby reducing health inequities.

The recent Creative Health Review by the All-Party Parliamentary Group (APPG) on Arts, Health and Wellbeing and the National Center for Creative Health (20) outlined how creativity – including creative activities in nature – is fundamental to supporting healthier, happier, and economically flourishing communities and creative health should therefore be integrated into a whole system approach to health and social care. The report makes various recommendations that build on the existing foundations and policy drivers to achieve their vision for creative health (p.13):

*Central to this vision will be the development of person-centred and community-led approaches, informed by lived experience, which will mobilise existing creative, cultural and community assets in order to best meet local need and reduce inequalities.*

Mobilizing existing creative, cultural and community assets is central to asset-based community development and other asset-based approaches that are gaining credence in United Kingdom public health policy making [e.g., (21, 22)]. Such approaches seek to identify the assets, skills and capacities that already exist within communities – rather than deficits and needs – so as to promote resilience, independence, and wellbeing by focusing on what can be strengthened through communities working together (21). Assets may include the gifts, skills, and capacities of individuals; informal citizens associations (e.g., religious, cultural, recreational); and formal institutions within the local community (23). Schools have been identified as key institutional community assets that can promote wellbeing for children, the wider school, and the local community and ultimately contribute to overall public health (22, 24). The importance of schools is also recognized in the Healthy Child Programme (25), the national prevention and early intervention public health framework for children, young people, and their families in the United Kingdom with the ethos: “universal in reach and personalised in response” (26). Integrating education, health, and social care as well as third sector and voluntary services, the Healthy Child Programme takes a place-based approach drawing on local community assets and developing local solutions to address public health challenges (26, 27). This is operationalized through a schedule of interventions (28); for improving children’s mental health specifically, these interventions include taking action to “promote and support the development of a whole-school approach” and “use the assets that contribute to positive health and wellbeing” (n.p.). Within the former, activities that enhance social skills and confidence including art groups are recommended, while the latter suggests drawing on community members, social networks, and local community groups; physical, environmental, and economic resources; and public, private, and voluntary organizations and services (28).

Furthermore, the United Kingdom government has endorsed a whole-school approach to mental health (7) that considers how all aspects of school life can influence mental health, including: leadership and management, curriculum teaching and learning, student voice, staff development, identifying need and monitoring impact, working with parents and carers, targeted support, and ethos and environment (29). For example, the National Institute for Health and Social Care Excellence (NICE) guideline [NG223] (30) on social, emotional, and mental wellbeing in primary and secondary education recommends adopting a whole-school approach for the wellbeing of children, young people, and staff, and ensuring a culture, ethos and practice that strengthens relational approaches, inclusion, and psychological safety. Among other things, it suggests the following to support a whole-school approach:

- *Having an outward-facing approach to the community and to engaging with local communities and groups.*
- *Strengthening links to external agencies that can provide additional support, such as local children’s health and care services and relevant voluntary and community sector organizations.*
- *Involve parents and carers in designing and implementing the whole school approach.*

Such policies and recommendations position schools as a community asset for children’s mental health and wellbeing and thus overall public health; however, it is the individuals within and connected to the school community that are often the driving force. This was elucidated in a systematic review by Herlitz et al. (31) that explored the sustainability of public health interventions in schools after initial funding and/or resources end. The review found two key facilitators: committed senior leaders that could support and integrate the intervention into the school, and school staff who were confident in delivering the intervention and believed in its value.

Further to this, schools with strong social links and support from parents/carers were more likely to be motivated to develop and maintain public health interventions (31). Therefore, one way of building capacity for schools to implement and sustain public health interventions would be to mobilize community assets such as parents/carers and other community members such as local volunteers. Volunteering is defined as “someone spending time, unpaid, doing something that aims to benefit the environment or someone who they are not closely related to” (32). Volunteers are an essential human resource, supporting local cultural and community arts projects and sustaining the wider cultural sector (33, 34). The voluntary, community and social enterprise sector has been recognized as a vital partner in delivering public health interventions (35). Moreover, volunteering has been advocated as a public health intervention itself as a way of engaging people in their local communities to build social capital and enhance the mental health and wellbeing of volunteers (36). Despite the nature of volunteering changing in recent times due to factors such as generational attitudes, increased use of technology, austerity, and the COVID-19 pandemic (37–39), the opportunities for mobilizing volunteers in delivering arts-in-nature practice hold great potential for promoting reach and sustainability.

Within this context, this article reflects on the ‘Branching Out’ research, which set out to investigate how an established arts-in-nature program called ‘Eco-Capabilities’ could be scaled up from time-limited projects involving small numbers of children, to a sustainable public health intervention involving whole-school communities. Eco-Capabilities intended to respond to concerns around children’s wellbeing; their disconnect with the environment; and a lack of arts engagement within the school curricula. Drawing on Amartya Sen’s theory of human capabilities as a proxy for wellbeing (40), ‘eco-capabilities’ refers to how children define what they feel they need to live a fully good human life through environmental sustainability, social justice, and future economic wellbeing. The Eco-Capabilities program engaged a total of 101 children aged 7–10 in eight full days of arts-in-nature practice called ‘Artscaping’. Developed and delivered by project partners Cambridge Curiosity and Imagination (CCI),^1^ Artscaping holds three key characteristics: “to affect and be affected by arts, nature, place and space; to create a response from materials and feelings to express new ideas; and to enhance the environment in ways that delight” (41), p. 5. A wide range of activities – such as drawing what they could hear, collecting natural materials to inspire and create artwork, exploring different natural environments and reflecting on colors, textures, and patterns – encouraged children to slow down and use their creativity and imagination to tune into nature. Research by Walshe et al. (41) alongside the Eco-Capabilities program identified four pedagogical elements:

- *Extended and repeated arts in nature sessions.*
- *Embodiment and engaging children affectively through the senses.*
- *‘Slowliness’, which envelops children with time and space to (re)connect.*
- *Thoughtful practice, which facilitates emotional expression.*

These elements facilitated the development of eight (eco-)capabilities: autonomy; bodily integrity and safety; individuality; mental and emotional wellbeing; relationality: human/nonhuman relations; senses and imagination; and spirituality. Importantly, nature became more visible to children and its value was recognized as part of their ‘happy place’ and within the purview of wellbeing (41, 42). However, extending the reach of Artscaping to more children and ensuring its sustainability beyond projects that are restricted by funding, time, and resources remains a key challenge. Accordingly, the Branching Out model was developed by CCI and research partners from University College London and Anglia Ruskin University, in collaboration with charity Cambridge Acorn Project (CAP)^2^ who provide therapeutic services for children and families, along with children and young people’s mental health consortium, Fullscope.^3^ The Branching Out model intended to extend the reach of Artscaping by mobilizing community assets – including school staff and volunteers – as ‘Community Artscapers’ to implement its delivery and promote the mental health and wellbeing of children in primary schools.

## 2 Materials and methods

### 2.1 Branching out research overview

The Branching Out research methodology drew on Creswell and Plano Clark’s (43) exploratory multi-level mixed methods approach to investigate how CCI and partners could adapt their Artscaping practice for a model using Community Artscapers to reach more children and consider how this new model of delivery might fit within the existing health ecosystem. The first phase of the research was concerned with developing the Branching Out model. This included interviews with artists and school staff involved in the Eco-Capabilities program; a national online survey of arts organizations delivering arts and nature activities in schools; an e-Delphi Study with primary school staff with responsibility for children’s mental health and wellbeing; and stakeholder workshops including representatives from the health sector, local authority, education, and voluntary organizations. Findings from the first phase are reported elsewhere (44, 45). The second phase of the research reported here focused on the implementation of the Branching Out model across six pilot sites. The interdisciplinary research team are based within the social sciences with expertise and experience across education, health, sustainability, and social policy. The last author and Principal Investigator worked previously in secondary schools with a background in geography before focusing on higher education and research into climate change and sustainability. The third author worked previously in primary schools as a teacher and inclusion manager, responsible for coordinating and implementing health and education interventions. The first and second authors have experience in researching the relationships, infrastructures, and processes involved in implementing participatory arts programs for various communities across different health, care, and education settings. Ethical approval for the research was granted by the University College London Institute of Education Research Ethics Committee. All participants were fully informed about the research using the appropriate participant information sheet and signed a consent form before data collection.

### 2.2 Branching out model

After the first phase of the research, the Branching Out model began to take shape with the following components: recruiting Community Artscapers, training Community Artscapers, selecting children, Artscaping sessions, and reflecting on Artscaping sessions. Delivery of the pilot was led by CCI and CAP with support from Fullscope and the research partners. It is important to note that the model was still in development during the pilot, so although suggestions were made regarding each of the components, CCI and CAP worked closely with each school to adapt the model for their particular context and children. Schools were responsible for recruiting Community Artscapers, though CCI developed a role description and volunteer policy, advertisements, and a handbook, and offered additional support around issues such as inclusive recruitment strategies and screening processes. Community Artscapers then attended a training day delivered by CCI and CAP covering the purpose of Artscaping and the Community Artscaper role, how children might benefit from it, the outdoor spaces that might be utilized, and how to organize and deliver Artscaping sessions. The training resources had been co-created by CCI artists with children from a local primary school who were already engaged in Artscaping.

Based on findings from the first phase, it was suggested that volunteers should work alongside school staff either with small groups of children or with the whole class, with Artscaping sessions taking place once a week for at least 1 h in the afternoon (45). The training encouraged Community Artscapers to hold these sessions outdoors in nature, starting with a grounding activity that supported children to engage their senses before moving into more focused creative activities that facilitated connection with the environment, and ending with a reflection on the session. The training resources included various ideas for activities such as those used in Eco-Capabilities, but Community Artscapers were also encouraged to create their own activities reflecting on what worked well in previous sessions and following the interests of the children. The resources also included a ‘Companionship Compass’ that had been co-created with children and reminded Community Artscapers to be kind, hold back, celebrate, and listen and respond during Artscaping sessions. A member of the CCI and/or CAP team attended the first session in each school to support Community Artscapers and the CCI Project Coordinator maintained contact throughout and offered one-to-one support as needed.

### 2.3 Piloting the branching out model

The Branching Out model was piloted in six primary schools geographically dispersed across both rural and urban settings of Cambridgeshire in areas of high disadvantage, that is areas with an IDACI of 4th or 5th quintile (the Income Deprivation Affecting Children Index, which measures the proportion of children living in income deprived families). The initial intention was for all schools to recruit local volunteers to be Community Artscapers working with a small group of eight to 12 children, supported by school staff; however, some schools found volunteer recruitment challenging and/or felt that including staff would lead to greater sustainability. In one school Artscaping was facilitated entirely by a group of four volunteers with a small group of children. In three schools, a range of school staff – including teaching assistants, teachers, and pastoral leads – worked with volunteers who were usually parents of children in the school to facilitate Artscaping with small groups of children. In two schools, Artscaping was facilitated entirely by school staff: in one of these a range of school staff worked with a small group of children and in the other the class teacher worked with half the class (a larger group of children) alternating each week. All adults involved in facilitating Artscaping were considered Community Artscapers.

The process of selecting children to participate was different in each school, but usually involved consultation between the senior leadership and teachers through pupil progress meetings and discussions with special educational needs coordinators (SENCOs) and pastoral leads. Artscaping was seen by the schools as an opportunity to support those who had been identified as needing extra support due to concerns about their mental health and wellbeing but were not eligible for external mental health support. In each school, Community Artscapers delivered 1.5-h Artscaping sessions with children for eight weeks in Autumn 2022. Researchers attended one session in each of the schools to inform subsequent data collection.

### 2.4 Pilot data collection

All school staff and volunteers involved in the Branching Out pilot were invited to attend an online semi-structured interview that took place a few days after the final Artscaping session, though due to busy schedules not everyone was able to participate. Those working closely together at the same school often opted to do the interview together so they could collectively reflect on their experiences, resulting in a mixture of four group interviews (including nine participants, mostly volunteers) and six individual interviews. Across the 10 interviews, participants included 11 Community Artscapers, comprised of six school staff (four teaching assistants, one teacher, one pastoral administrator) and five volunteers (who were mostly parents of children in the schools, though not children taking part in the pilot), as well as four members of school senior leadership (three headteachers, and one executive headteacher across two of the pilot schools).

The interviews encouraged participants to reflect on their experience of implementing the Branching Out model and facilitating Artscaping. The same questions were asked across individual and group interviews, adapted as appropriate for volunteers, school staff, and school senior leadership. Questions asked about their understanding of the approach and why they were interested in it; their experience of the training day; the applicability of the model for the school; their perceptions of the impact on the children, the school, and on themselves personally; how the children had been selected; how Community Artscapers had been recruited; their experiences of working with school staff/volunteers (as appropriate); any challenges encountered; any future plans for continuing; and any suggestions for improvement.

The Director and Project Coordinator from the CCI team were also interviewed, focusing on the process of establishing and supporting the Branching Out model in schools. This interview took the form of a reflection whereby the team were asked to reflect on the process of the setting up and managing Artscaping in the different schools, the training provided for Community Artscapers, and the ongoing support provided to the schools by the team during the Branching Out pilot. All interviews ranged from 45 to 75 min long and were audio recorded and professionally transcribed.

### 2.5 Pilot data analysis

Transcriptions were subject to reflexive thematic analysis guided by Braun and Clarke (46–48), which took an inductive, semantic, and critical realist approach to understanding the qualitative reflections, perspectives, and experiences of participants. All members of the research team were involved in the process of analysis, first through familiarization with the data individually before collectively discussing potential codes and themes. The first and third author then undertook an iterative process of coding the data and came together to generate initial themes. The themes were reviewed and further developed by all members of the research team, drawing on our interdisciplinary background and experience to identify key concepts and patterns of meaning. Based on this, the first and third authors refined and defined each theme and wrote up the findings.

The findings have been used to produce a practitioner guide ‘Artscaping: A guide to establishing arts-in-nature opportunities in your school’ underpinned by ‘Artscaping with Community Artscapers: Research findings from the Branching Out project’.^4^ The findings presented in this article provide insight into the potential of the Branching Out model in mobilizing community assets to support the mental health and wellbeing of children in schools. Quotes are used from a range of participants to illustrate the themes; however, they are credited broadly to the roles of ‘Volunteer Community Artscaper’, ‘School Staff Community Artscaper’, or ‘School Senior Leadership’ to protect the anonymity of participants who may otherwise be identifiable due to the varying configurations of delivery across pilot schools.

## 3 Findings

### 3.1 Mobilizing community assets

The overarching theme mobilizing community assets is comprised of sub-themes: framing the opportunity, recruiting and sustaining volunteers, training and supporting Community Artscapers, and tensions in roles and responsibilities.

#### 3.1.1 Framing the opportunity

One of the first steps in mobilizing community assets as Community Artscapers was to develop and conceptualize the opportunity in a way that would engage individuals and utilize their gifts, skills, and capacities as assets to become the driving force in delivering the Branching Out model. The Community Artscaper role was framed as an opportunity for school staff and volunteers to engage and interact with children in a novel way, outside of the classroom, fostering a connection with nature using arts and creativity. Involvement in the pilot held impacts for the children, but also for the Community Artscapers themselves, and the wider school community, demonstrating the potential for this model as a public health intervention.

Effective communication of the role description was essential to underscore the core responsibilities, practicalities, and requisite attributes for potential Community Artscapers. Participants in the pilot shared a profound passion for arts, being outdoors, working with children, and supporting their mental wellbeing, as highlighted by one volunteer.

*…it just encompasses a lot of my interests. So I thought it was just a really valid thing to be a part of… and I suppose my skill sets because I do a lot of outdoor things and I’m an art teacher, so it kind of married the two nicely together.* [Volunteer Community Artscaper].

Importantly, the role did not necessitate specific artistic skills so as not to discourage potential volunteers who may perceive themselves as *“no good at art.”* Instead, the emphasis lay on the ability of Community Artscapers to listen and to provide a non-judgmental and creative environment for children to freely express themselves through the exploration of art materials and the natural surroundings.

#### 3.1.2 Recruiting and sustaining volunteers

While school staff were often designated as Community Artscapers by the headteacher, for most schools recruiting and sustaining volunteers was crucial in mobilizing community assets for the Branching Out pilot. After a process of research and development with schools, CCI developed a volunteer policy (for schools that did not have one already), advertisements, a handbook, and offered additional support as required. Schools recruited volunteers through newsletters, emails, social media, noticeboards, and word-of-mouth communication. School leadership advocated for a “*direct appeal*” to engage parents and carers of children within the school, underscoring the training component as a key benefit.

*…the great thing about this project is it did have a training session which was lovely because that meant you could sort of get the people and say well you know this training would really help you be skilled up in it and they could all go together to the training and meet each other.* [School Senior Leadership].

Although a few schools were nervous about getting the right adults to work with children with specific needs, many participants emphasized the significant value that volunteers can bring to the children’s Artscaping experience, particularly in the relationships that some volunteers were able to cultivate with the children. Working with volunteers was also important for strengthening connections between the school and the local community. For the most part, school staff celebrated the contribution made by volunteers.

*…they are ever so grateful at school as well, they are very appreciative of us going in and helping and they tell us that quite a lot… it’s not just the direct teacher… who says thank you, it’s, you know, everyone, all the other teachers… they are really grateful.* [Volunteer Community Artscaper].

This was particularly important for sustaining volunteers, in terms of them feeling valued and that their contribution was meaningful and appreciated. It also demonstrates the recognition of how community volunteers can become part of the whole-school approach.

#### 3.1.3 Training and supporting community Artscapers

A pivotal component of the Branching Out pilot was the support provided by CCI and CAP. A training day allowed volunteers and school staff to hear about the purpose of Artscaping and the Community Artscaper role, understand which children might benefit from it and the outdoor spaces they might utilise in their schools, and learn how to organize and deliver Artscaping sessions. It also offered the opportunity for Community Artscapers to experience the activities for themselves, enabling a better sense of the potential impact on children’s mental health and wellbeing.

*So the first part of the training… they did some Artscaping with us… and that was really helpful to see… how calming it can be, but also the effect that it can have mentally on children who are stressed and… have lots of things going through their head.* [Volunteer Community Artscaper].

It additionally served as a source of inspiration, enthusiasm, and excitement, gave volunteers and school staff the opportunity to meet and begin planning their sessions, and established a network of Community Artscapers across different schools. This facilitated the exchange of ideas, practices, and enthusiasm among volunteers, school staff, and schools.

*Everyone was very open to talking… it was really lovely to hear people’s experiences of their pupils and how things would work and also their ideas… You could see this sort of inspiration spreading among everybody… it was useful for networking…* [School Staff Community Artscaper].

Some participants with experience of working in schools felt that the training may be insufficient for volunteers who did not have experience in structuring or delivering sessions. At the same time, it was acknowledged that even the most comprehensive training could not prepare Community Artscapers for all eventualities. Accordingly, CCI and CAP provided ongoing support, including resource provision, presence during the first sessions, and responsive assistance when issues arose. This support was appreciated and central to the experience of Community Artscapers and schools.

*…when I put my thoughts and feelings out there, it’s been listened to, and we have had offers of help… I felt supported throughout it.* [School Staff Community Artscaper].

It was suggested that a resource bank of different ideas for sessions and activities could be developed with the potential for online sharing across different schools. One school had limited access to nature, but recommended ways of being creative with their outside space, using different areas of the school or neighboring grounds, and bringing in natural resources collected elsewhere. In addition, a WhatsApp group for pilot participants enabled the sharing of ideas and activities.

*What I have seen through the WhatsApp is actually the sharing of good practice across schools and… inspiring people’s ideas… you think, you know, that could work for us and we could do that… it makes you feel then you are part of a network of schools doing this rather than just doing your own practice in your own school…* [School Senior Leadership].

Ongoing training was identified as essential to ensure the sustainability and continuity of the program by renewing enthusiasm and bringing in new school staff and volunteers. Suggestions from participants included a longer training program, potentially delivered online or with an ongoing online element, and the possibility of a ‘train the trainer’ program, though there were also concerns that this might dilute the training experience. Certainly, training and supporting volunteers would be a key component in mobilizing community assets to increase capacity for delivering Artscaping for children in primary schools.

#### 3.1.4 Tensions in roles and responsibilities

Mobilizing community assets required a coordinated approach to ensure clear roles and responsibilities in delivering Artscaping for children in primary schools. Tensions around the roles and responsibilities of school staff versus volunteers arose during the pilot. Some participants felt that the program needed school staff to take the lead to ensure there was always someone driving it and address potential issues around volunteer commitment and experience. This was echoed by a member of senior leadership who suggested using teaching assistants would be an easier option than recruiting volunteers.

*I would just say get a few volunteers, you know ideally. I do think you probably need two people in school who were there all the time to help take an ownership of it… leading it within the school… because it does need the school to really want to do this and drive it as well.* [School Staff Community Artscaper].

However, some volunteers felt superfluous: with too many adults and school staff leading they felt more like participants than facilitators. This undermined their aspirations to make a meaningful difference and a useful contribution to the school. It also contradicted their expectations from the training day of being involved in the organization and delivery of the sessions, and impacted on their overall perception of the program and whether they would continue volunteering as Community Artscapers.

*…I think [volunteering] once was enough probably… I’m not sure how much I added to it… apart from being an extra adult… I was the only one who did not know [the children]… I was definitely the least useful member of the team.* [Volunteer Community Artscaper].

There were also tensions around volunteers being “*outsiders*,” in terms of their limited familiarity with the children, concerns around safeguarding training, knowing the school layout and how to access resources, and balancing expectations alongside school staff in a non-remunerated role. In some instances, essential information was not communicated to volunteers in a clear or timely manner. As a result, there were suggestions to establish greater structure across the program so that roles and responsibilities could be discussed and agreed in advance, and effective communication was highlighted as crucial.

### 3.2 Impacts for children

Participants spoke about their perceptions regarding the impacts for children of taking part in the Branching Out pilot, including discussions around: mental health provision, freedom in creativity and being outside, personal development, emotional impacts, and social connection.

#### 3.2.1 Mental health provision

The impact on children meant that Artscaping delivered by Community Artscapers could serve as a form of “early intervention” or another “wave” of support for emerging mental health concerns before the point of being eligible for other provisions.

*We have identified a number of pupils who we were concerned had sort of emerging mental health difficulties… to get external support, it is quite difficult… but then you want to tackle these things sooner rather than later so that they stop developing further… there was a gap in the provision that we could offer and we could access… So when I heard about this project I thought, that… could be a really useful thing to help support those children.* [School Senior Leadership].

This observation demonstrates the potential impact of Community Artscapers delivering Artscaping as a public health intervention through a whole-school approach to meet a gap in mental health provision. Importantly, it helped schools to think about how they could offer different forms of support that align with the discourse around mobilizing existing creative, cultural and community assets.

*…it’s made me think a bit differently about what’s possible in terms of the provision and the support that can help young people, that mental health support does not always have to be something that’s intensive, something that’s gone through referral processes, that relies on external agencies, that relies on a huge amount of funding.* [School Senior Leadership].

#### 3.2.2 Freedom in creativity and being outside

The freedom of Artscaping was emphasized as fundamental to the Branching Out model, allowing children to follow their creative instincts and initiative. Using nature as a starting point tapped into their imagination, and children were free to enjoy the process of making and creating without the expectation or pressure to produce a particular outcome.

*I think it was that they did not have to produce a certain piece of work. It was very much free flow, as little, as much as you want in any way you want. No boundaries… there is not a result. Because there’s too much focus on results in school. It’s all about you need to have got to this point, if you have not got to this point you are not very good. And it wasn’t like that.* [Volunteer Community Artscaper].

It also meant utilizing the natural outside environment as an asset in supporting children’s mental health. The freedom associated with being outside was fundamental to Artscaping, as one participant articulated “*it did make a difference… there is something special about being outside*.” Being outside brought out a different side to the children – and the adults – and fostered experiences and encounters that helped children to connect with nature in new ways.

#### 3.2.3 Personal development

Artscaping fostered children’s confidence in terms of participating, speaking, taking ownership of what they were doing and what they wanted to do, and developing a sense of achievement.

*…the ability of some of those children, you know, they are not going to be the top scorers [academically], but they were achieving every week. They were achieving something that they were incredibly proud of… in a classroom setting they often do not get to achieve, or they do not feel like they have achieved, because… compared to everyone else… It was that sort of release.* [Volunteer Community Artscaper].

The consistent structure of the Artscaping sessions had a positive impact on children’s ability to focus, interact, and participate. It was reported that the children also became more confident back in the classroom in their creativity, curiosity, and were engaged and ready to learn.

*…if you have a child who’s now a much more engaged learner because they are gaining the mental wellbeing from participating in something like the Artscaping you can then see that coming through in their work as well because they are more engaged in what they are doing generally.* [School Senior Leadership].

#### 3.2.4 Emotional impacts for children

Artscaping appeared to have a positive impact on the emotional wellbeing of the children, as Community Artscapers reported their enjoyment and excitement, but also how the sessions instilled a sense of calm.

*…they might be feeling miserable about the rest of the day or what’s going on at home but at that moment in time, when I’m asking them about how they are feeling, they are saying, “happy,” they are happy… they all say, “oh, calm and peaceful and happy.”* [School Staff Community Artscaper].

Children were able to focus on the creative activities; this sometimes translated into a physical response with one participant reflecting: “*you could almost see them physically relax when they came in*.”

#### 3.2.5 Social connection

During Artscaping sessions, children had new opportunities to connect with adults with the potential to promote intergenerational and community cohesion. For example, the Artscaping approach of “*working alongside*” helped children to build relationships with volunteers and see teachers in a different context outside of the classroom and on a more equal footing.

*Everybody got stuck in and made something alongside each other… that was really lovely as well because… I think it gave the children more freedom because then it was a unanimous shared experience and I think that contributed to them feeling really at ease and relaxed.* [Volunteer Community Artscaper].

The sessions also facilitated connections among children, especially in mixed-age groups that do not typically interact, and new friendships developed across year groups as a result.

### 3.3 Impacts for Community Artscapers

Contributing to the program also had an impact for the Community Artscapers discussed under the following sub-themes: Artscaping practice making a difference, emotional wellbeing, personal and professional development, and connection and community.

#### 3.3.1 Artscaping practice making a difference

Community Artscapers were new to the practice of Artscaping and appreciated the freedom to follow the children’s lead without concerns around ensuring they produced a certain outcome. They appreciated the opportunity to be outside, be creative, and connect with nature for themselves, demonstrating the value of volunteering and being a part of the pilot. This also helped them to recognize how important and valuable Artscaping practice was within the context of the curriculum and wider education system.

*It’s just seeing children be able to come out and have a conversation, and to see the effects that nature, being outdoors has had on them and how it affects their work and their artwork and their interest…* [School Staff Community Artscaper].

Accordingly, knowing that Artscaping was making a difference for the children was a key impact, especially for volunteers as this was often a motivating factor for their volunteering. Meanwhile, seeing the benefits for children in relation to classroom skills was also important for school staff.

#### 3.3.2 Emotional wellbeing for adults

Community Artscapers reported a positive impact on their own emotional wellbeing derived from Artscaping. The freedom in creativity and being outside meant that adults were able to enjoy the time with the children, working alongside them and watching them experience Artscaping and its positive impacts.

*The pressure was off for an hour to just have a bit of fun and enjoy being with them.* [School Staff Community Artscaper].

The Artscaping experience was especially compelling for some Community Artscapers when it aligned with their personal or professional approach, passions, and lived experience, and these participants were particularly grateful for their involvement.

*I’m just a big believer of being outdoors… Because I know for myself… I actually suffer with anxiety myself, being outdoors it helps me… I wanted to roll that out with the children, and it’s had such a huge effect… it’s such a beautiful thing to be part of, I cannot even tell you…* [School Staff Community Artscaper].

This provides support for the idea of volunteering as a public health intervention itself by enhancing the emotional wellbeing of those delivering the Artscaping practice.

#### 3.3.3 Personal and professional development

Community Artscaping was seen as an opportunity for personal and professional development for both volunteers and school staff. The training fostered confidence and empowerment, allowing them to take ownership over the project. For some volunteers this was helpful in their preparation for returning to work after a career break, while for school staff it legitimized a different way of working with the children.

*…it gives you confidence to do something that I guess in some ways would’ve been instinct for me to do but I would never have been absolutely certain. I felt I’ve got backing, you know? It’s fine to go ahead and just let them express themselves and give them freedom… they are always saying that in school, “oh yeah, that’s great, you must do that,” but there’s always so many pressures to do other things.* [School Staff Community Artscaper].

Additionally, parent volunteers reported being proud of their involvement and being able to apply the Artscaping approach to other areas of their lives, again supporting the wider school and community benefits of Artscaping beyond children’s mental health.

*So it’s really made me think about… my own practice working with children and the kind of transferable kind of learning from that really, about understanding that children aren’t going to share with you immediately, kind of, what’s going on for them and that they need that time to build trust and kind of have the flexibility.* [Volunteer Community Artscaper].

#### 3.3.4 Connection and community

Building connection and community was a key impact from Community Artscaping. Both volunteers and school staff expressed their passion for working with children and the opportunity to develop meaningful connections with them through Artscaping.

*…caring for these children that need a bit of extra support, you know, [I] really felt that responsibility, but I always enjoyed trying to do something positive with children. So I really felt the, I guess the satisfaction of that.* [Volunteer Community Artscaper].

For school staff this was particularly pertinent as the Artscaping sessions allowed them to work with a smaller group and encourage freedom in creativity and being outside, which strengthened their relationships. Additionally, bringing volunteers into the school helped them to feel part of the school community. This theme in particular exemplified how Community Artscapers delivering Artscaping for children exemplified a whole-school approach.

## 4 Discussion

The Branching Out model represents an innovative way of extending the reach of Artscaping by mobilizing community assets and thus demonstrates potential as a public health intervention to support the mental health and wellbeing of children in primary schools. The findings around mobilizing community assets align with the numerous public health policy drivers set out in the introduction to promote children’s mental health. In particular, the whole-school approach and connecting with the local community were integral to the establishment and delivery of Artscaping with Community Artscapers as community assets, and to generating positive impacts for both children and Community Artscapers.

Artscaping with Community Artscapers in schools exemplifies the integral role of schools as institutional community assets (22, 24). Not only were they the host of the pilot, but schools were also essential in framing the Community Artscaper opportunity to engage school staff and members of the local community, recruiting and sustaining volunteers, and supporting the Community Artscapers. These factors facilitated the mobilization of individuals both within the school, which has been shown to be a key facilitator in the sustainability of public health interventions in schools (31), and from the local community, which has been identified as vital to delivering public health interventions (35). Furthermore, it promoted a whole-school approach (30) by engaging parents, carers, and the local community in delivering Artscaping, as well as strengthening links with local community organizations and outside community spaces. This resonates with work by March et al. (49) who recommended a whole-school approach to mental health and that for school leaders to create a sustainable and healthy ecosystem for mental health and wellbeing, it is necessary to harness the support of the wider community, including families and local resources.

Taking on the Community Artscaper role often aligned with personal or professional interests and included training and support that fostered feelings of investment in the Branching Out pilot. As a development opportunity for school staff, the model promotes wider ambitions such as maximizing the impact of teaching assistants (50), particularly in delivering high quality and evidence-based structured interventions outside of the classroom (51). This is particularly important as training and career progression opportunities for teaching assistants are limited and generally the responsibility of individual schools (52). Meanwhile, for volunteers the opportunity added value for individuals both in terms of development and being able to make a difference. Indeed, the ‘Time Well Spent’ national survey of 10,203 people found that wanting to improve things or help people was the most common reason for volunteering, but other motivations included wanting to use or improve existing skills or gain new skills, and training was regarded positively by those who received it (53). It is important to acknowledge the funding that would be needed to underpin the training and development required for the Branching Out model. However, at the same time as benefitting individual school staff and volunteers, such investment in the development of Community Artscapers would contribute to building the capacity for schools to implement and maintain public health interventions (31).

Indeed, recruiting school staff and volunteers as Community Artscapers represented an important opportunity for utilizing the untapped potential of assets within communities to support the mental health and wellbeing of children. The need for prevention and intervention initiatives in schools to support children’s mental health has increased exponentially since the pandemic with both an increased number of children needing support and the challenges of mental health provision through the NHS (8–10). However, the systematic review by Herlitz et al. (31) found various contextual barriers to the sustainability of public health interventions in schools after initial funding and/or resources end. At a school level these included insufficient funding and/or resources, staff turnover and a lack of ongoing training, and the norm of prioritizing educational outcomes under time and resource constraints. In a later systematic review, March et al. (54) also identified school culture and school leadership as barriers to sustainability at a school level. In addition, there are wider system level barriers to sustainability of public health resources in schools including shifting priorities and turnover of key personnel (54). Accordingly, the Branching Out model enables not only the training and impetus that can often be a barrier, but also offers a pathway to increasing capacity and sustainability through community volunteers.

Although volunteering surged during the pandemic, there has since been a reduction in the numbers of people volunteering compared to pre-pandemic (55). This was observed by the CCI team as schools have had to focus on core delivery and reported that many of their previously regular volunteers had fallen out of practice while others no longer felt comfortable within the school environment with COVID-19 still a risk for certain groups. However, the Branching Out model offers new possibilities for reigniting community engagement and strengthening communities. By bringing people together for a common purpose, volunteering can build a sense of self but also connection to others, cohesive relationships, and a sense of belonging and community (56, 57). Certainly, the training established a network of Community Artscapers across schools, as well as a core group for delivery within schools, and generated positive impacts around connection and community. While there were tensions in roles and responsibilities regarding the delivery of Artscaping, such issues would be easily addressed by greater structure and communication underpinned by learning from the pilot. Despite these positive reflections, there are wider concerns within the discourse on mobilizing community assets and particularly volunteering. For example, the ideological and social policy drivers behind asset-based community development have been accused of being “a capitulation to neoliberal values of individualization and privatization” (p.430) thus presenting dilemmas for community development (58). Nonetheless in the current context, through community mobilization the Branching Out model has the potential to address critical gaps in both the education and healthcare systems in terms of supporting children’s mental health, with clear positive impacts for children but also for Community Artscapers and the whole-school community.

This research did not set out to measure the impact of Artscaping on children, Community Artscapers, or schools specifically; however, it was clear from the interviews with volunteers and school staff that introducing Artscaping into the schools was perceived to have a positive impact. This echoes the findings from the Eco-Capabilities program and other research reporting on the impact of arts-in-nature practice on children (19, 41, 42). What is noteworthy, however, is the reported impacts on the Community Artscapers themselves, including: making a difference, wellbeing and emotional impacts, personal and professional development, and connection and community. This mirrors existing literature that reports on the impact of volunteering for those who volunteer. For example, in the ‘Time Well Spent’ survey (53) most people reported a sense of personal achievement and feeling they make a difference, however, satisfaction with volunteering was associated with feelings of support, recognition and belonging. People tend to volunteer for altruistic reasons but if the activities are not valued by the volunteers, they are unlikely to provide benefits to the volunteers (36). In the current research, Community Artscapers observed benefits to the children taking part and therefore it is likely this would have contributed to their own sense of wellbeing, particularly as it has also been noted that the positive impact of volunteering on quality of life is dependent on reciprocity (36).

The findings are important as they demonstrate the potential of mobilizing community assets in the form of schools and volunteers to support the wider public health agenda, with arts-in-nature practice having an impact on the children, school staff and volunteers, and the wider school. In the introduction, the notion of schools as community assets was outlined, along with the whole-school approach in supporting the mental wellbeing of children, young people, and staff. The training and utilization of Community Artscapers fits the NICE guideline [NG223] (30), which calls for schools to engage with local communities, groups, and other external agencies. It also sits within the first level of the Healthy Child Programme framework where community assets are seen to support health and wellbeing (25–28), not only in the development of the children’s mental health and wellbeing but also that of the school staff and volunteers themselves as Community Artscapers and in the community setting of the school. The Branching Out model provides the means for schools to identify children who need low level emotional health and wellbeing support, and to maximize the role of Community Artscapers to facilitate sessions with these children. This would complement recent policy initiatives and guidance with the development of Mental Health Support Teams (MHSTs) in education settings from a health perspective (59), and pressure to have a school staff member trained as a senior mental health lead from an education perspective (60). While these supports are clearly needed, the Branching Out model can complement such interventions and fit into whole-school approaches to promoting children’s mental health and wellbeing (61). Schools and local communities can provide an environment to promote good mental health through developing social skills, socio-emotional competencies and learning outcomes (62). Within the current context of barriers to accessing professional help for mental health (8–10), there is an urgent need to provide early interventions for children experiencing poor mental wellbeing before the need for referral to local health teams, such as the school nursing service or the NHS Child and Adolescent Mental Health Services (CAMHS).

In terms of limitations to the research, the sample size is modest due to the research being a pilot for the implementation of the new Branching Out model of delivery in six primary schools. The participants reported observing positive impacts on the children involved, but the research did not capture the views of the children or measure any potential changes in children’s wellbeing directly. As reported in the introduction, previous research has demonstrated the impacts of Artscaping, but the perspectives of children on the Branching Out model using Community Artscapers have not yet been researched. It is essential that children’s voices are incorporated into the public health policy agenda because they are the central stakeholder in wellbeing provision in education (63). Future research should include the opportunity not just to evaluate the impact of Artscaping on the children but also engage children as part of the research process. Such research should ensure methods adopted are age-appropriate and accessible and enable all children to contribute their perspectives; creative and participatory research methods are prominent examples that are increasingly used within education to enable children’s voices to be heard (64). Additionally, it would have been useful to explore the barriers to recruitment of local community members as volunteers, but when the pilot was planned it was not anticipated that recruitment would be a particular issue. While the research demonstrates the potential to roll out the model more widely, the findings could be used to inform the further refinement of the Branching Out model and further research to strengthen the evidence for effective implementation in the future.

## 5 Conclusion

This paper presents the findings from a new and innovative model that engaged school staff and volunteers in delivering arts-in-nature practice to children in primary schools to promote their mental health and wellbeing. Despite the public health policy drivers around mobilizing community assets and focusing on schools as key institutional community assets, little research has looked at this in practice particularly in the current context since the COVID-19 pandemic. The findings indicate how the Branching Out model could provide an opportunity for schools to develop their staff, recruit and support volunteers from the local community, and increase their capacity to promote the mental health and wellbeing of their children. The positive impacts for children, volunteers, school staff, and the wider school community are compelling. Given this was pilot research, the wider public health impact and sustainability of the Branching Out model is yet to be seen. However, through community mobilization there is potential for the Branching Out model to become replicable and self-sustaining across schools to promote children’s mental health as a public health intervention.

## Data availability statement

The datasets presented in this article are not readily available because of confidentiality to protect the identity of participants in this small-scale qualitative research. Requests to access the datasets should be directed to hilary.bungay@aru.ac.uk.

## Ethics statement

This study involving humans was approved by the Institute of Education Research Ethics Committee, University College London. This study was conducted in accordance with the local legislation and institutional requirements. The participants provided their written informed consent to participate in this study.

## Author contributions

AD: Data curation, Formal analysis, Investigation, Writing – original draft, Writing – review & editing. HB: Conceptualization, Formal analysis, Funding acquisition, Investigation, Supervision, Writing – original draft, Writing – review & editing. FA: Data curation, Formal analysis, Investigation, Writing – original draft, Writing – review & editing. NW: Conceptualization, Formal analysis, Funding acquisition, Investigation, Supervision, Writing – original draft, Writing – review & editing.

## Footnotes

^1^ https://www.cambridgecandi.org.uk/

^2^ https://www.cambridgeacornproject.org.uk/

^3^ https://www.fullscopecollaboration.org.uk/

^4^ https://www.cambridgecandi.org.uk/our-work/schools/amplifying-artscapers
